# High potential for foliar water uptake in early stages of leaf development of three woody angiosperms

**DOI:** 10.1111/ppl.13961

**Published:** 2023-07-04

**Authors:** Adriano Losso, Birgit Dämon, Uwe Hacke, Stefan Mayr

**Affiliations:** ^1^ Department of Botany University of Innsbruck Innsbruck Austria; ^2^ Department of Renewable Resources University of Alberta Edmonton Alberta Canada

## Abstract

Foliar water uptake (FWU) is a widespread mechanism that may help plants cope with drought stress in a wide range of ecosystems. FWU can be affected by various leaf traits, which change during leaf development. We exposed cut and dehydrated leaves to rainwater and measured FWU, changes in leaf water potential after 19 h of FWU (ΔΨ), minimum leaf conductance (g_min_), and leaf wettability (abaxial and adaxial) of leaves of *Acer platanoides*, *Fagus sylvatica*, and *Sambucus nigra* at three developmental stages: unfolding (2–5‐day‐old), young (1.5‐week‐old) and mature leaves (8‐week‐old). FWU and g_min_ were higher in younger leaves. ΔΨ corresponded to FWU and g_min_ in all cases but mature leaves of *F. sylvatica*, where ΔΨ was highest. Most leaves were highly wettable, and at least one leaf surface (adaxial or abaxial) showed a decrease in wettability from unfolding to mature leaves. Young leaves of all studied species showed FWU (unfolding leaves: 14.8 ± 1.1 μmol m^−2^ s^−1^), which may improve plant water status and thus counterbalance spring transpirational losses due to high g_min_. The high wettability of young leaves probably supported FWU. We observed particularly high FWU and respective high ΔΨ in older leaves of *F. sylvatica*, possibly aided by trichomes.

## 1 INTRODUCTION

Foliar water uptake (FWU) is widespread in the plant kingdom and a potential mechanism of plants to cope with drought stress in a wide range of ecosystems (Binks et al., 2019; Hayes et al., 2020; Losso et al., 2021; Mayr et al., 2014, 2019; Roth‐Nebelsick et al., 2022; Schreel & Steppe, 2020; Schreel, Van de Wal, et al., 2019; Schreel, von der Crone, et al., 2019; Steppe et al., 2018; Waseem et al., 2021). In a recent review, Berry et al. (2019) reported that up to 85% of plant species can absorb water through their leaves. Atmospheric water, such as dew, rain or snow, may be crucial when soil water uptake is limited (Berry et al., 2019; Eller et al., 2013, 2016; Nadezhdina et al., 2010). In dry environments, leaf‐wetting events have been demonstrated to support water balance by suppressing transpiration (Alvarado‐Barrientos et al., 2014; Gerlein‐Safdi et al., 2018), and triggering FWU (e.g., Cavallaro et al., 2020; Eller et al., 2013). However, FWU can only occur when atmospheric water (liquid or gaseous) gets in sufficient contact with leaves (in terms of time and amount) and when a favorable water potential (Ψ) gradient drives the water flow into the leaf (and thus, in the opposite direction of transpiration; Eller et al., 2015). A decrease in leaf Ψ can be caused by limited soil water availability, gravitational forces due to plant height, and/or increased hydraulic resistance (Tyree & Zimmermann, 2002).

The four main recognized pathways for FWU are absorption (1) through the cuticle (Ketel et al., 1972), (2) by stomatal pores (Binks et al., 2020; Burkhardt, 2010; Guzmán‐Delgado et al., 2021), (3) by trichomes (i.e., uni‐ or multicellular epidermal leaf appendages; Ohrui et al., 2007; Schreel et al., 2020; Pan et al., 2021) and/or (4) hydathodes (i.e., microscopic pores situated typically found in the epidermis or leaf margin; Martin & Von Willert, 2000). The cuticle and open stomata are probably the most frequent pathways for FWU (Guzmán‐Delgado et al., 2021). Furthermore, trichomes, which are formed in the leaves of some species, can substantially contribute to FWU due to their structural and chemical design (Pan et al., 2021; Pina et al., 2016; Schreel et al., 2020, 2022; Waseem et al., 2021).

Leaf wettability is another crucial factor affecting FWU (Ali et al., 2022; Azad et al., 2015; Eller et al., 2013; Goldsmith et al., 2013; Gotsch et al., 2014; Gürsoy et al., 2017). It is substantially determined by the water‐repellency of leaf surfaces, which is increased by the presence of thick cuticles, cuticle chemical properties, waxes, and glabrous surfaces (e.g., Cavallaro et al., 2022; Kupper et al., 2006; Roth‐Nebelsick et al., 2022), while trichomes may decrease or increase the wettability of leaves (Fernández et al., 2014, 2017; Pan et al., 2021). Leaf structures and chemistry, and thus wettability, can vary within species and depend on leaf age (Boyce et al., 1991; Cape, 1983; Fernández et al., 2014; Kupcinskiene & Huttunen, 2005; Roth‐Nebelsick et al., 2022), leaf side (Brewer & Nuñez, 2007; Fernández et al., 2014), and environmental conditions (Aryal & Neuner, 2010; Goldsmith et al., 2017). Low leaf wettability has been shown to be advantageous in alpine environments, as it reduces the risk of dew on leaves and, thus, of extrinsic ice nucleation during cold nights (Hacker & Neuner, 2007, 2008; Wisniewski et al., 2002). High water repellency can also prevent contamination by dust and pollutants and even provide self‐cleaning properties due to the effective droplet run‐off (i.e., “Lotus effect”; Barthlott & Neinhuis, 1997; Neinhuis & Barthlott, 1997). In contrast, excess leaf wetness can promote the adhesion of pathogens and subsequent infections (Evans et al., 1992; Reynolds et al., 1989), but may also be advantageous for FWU.

During development, leaves undergo major changes in traits such as leaf area, leaf thickness and/or surface structures (e.g., cuticle maturation, trichome formation). In particular, the cuticle continues to develop and get thicker while hydrophobic waxes are synthesized throughout leaf expansion (Bukovac et al., 1979; England & Attiwill, 2011; Gratani & Bonito, 2009; Gülz et al., 1991; Varone & Gratani, 2009). Besides changes in leaf wettability, this leads to decreased cuticular conductivity and thus non‐stomatal transpiration (England & Attiwill, 2011) in older leaves, while unfolding and young leaves are limited in transpirational control. Accordingly, spring droughts may be particularly challenging for trees' fitness, and FWU of young leaves may be relevant to balance water losses. However, information on FWU during leaf development is still scarce.

In this study, we analyzed the potential for FWU, g_min_, and leaf wettability of leaves of three woody angiosperms (*Acer platanoides* L., *Fagus sylvatica* L., and *Sambucus nigra* L.) at three developmental stages: unfolding (2–5 days old), young (1.5 weeks old), and mature leaves (8 weeks old). We also measured the difference in leaf water potential (ΔΨ) before and after FWU. We expected differences (i) between leaf stages as young leaves are characterized by thin cuticles and immature stomata (e.g., incomplete closure, immature cuticles) and thus might be prone to higher transpiration but may be able to absorb more water (Bukovac et al., 1979; England & Attiwill, 2011; Gratani & Bonito, 2009; Gülz et al., 1991; Varone & Gratani, 2009), (ii) between species, due to the presence of trichomes in *F. sylvatica* and *S. nigra*, which may facilitate FWU in *F. sylvatica* (see Schreel et al., 2020), and (iii) between the wettability of adaxial and abaxial sides, also due to the presence/absence of both stomata and trichomes.

## 2 MATERIALS AND METHODS

### 2.1 Plant material

The study was performed on leaves of three woody angiosperms: *Acer platanoides* L., *Fagus sylvatica* L., and *Sambucus nigra* L. We compared unfolding leaves (2–5 days after bud break), young leaves (completely expanded but not finally ripened, ca., 1.5 weeks after bud break) and mature leaves (ca., 8 weeks after bud break). Accordingly, plant material was harvested between April 22 and May 16 (unfolding leaves and young leaves) and between June 14 and 16 (mature leaves) in 2021 and 2022.

From adult plants growing at forest sites near Innsbruck and Jenbach (Tirol, Austria), at least three 1‐m‐long branches (collected from different individuals) per experiment were cut, wrapped in dark plastic bags, and transported to the laboratory. Branches were re‐cut under water and rehydrated overnight in a water‐filled bucket, covered with a dark plastic bag.

### 2.2 Foliar water uptake

Rehydrated branches (with several leaves) were dehydrated on a bench. Leaves were cut at different intervals and water potential (Ψ) was determined with a Scholander apparatus (model 1505D pressure chamber; PMS Instrument, USA). As the petioles of *F. sylvatica* leaves were too short to be sealed in the lid of the pressure chamber, terminal end twigs with two or three leaves (main axes of ca. 3 cm) were used. *A. platanoides* and *S. nigra* had sufficiently long petioles for Scholander measurements. When Ψ dropped below −1 MPa, shoots were covered with a plastic bag for at least 15 min to avoid further dehydration. Leaves (*A. platanoides* and *S. nigra*) or end twigs (*F. sylvatica*) were cut from shoots, and Ψ was measured (−1.18 ± 0.02 MPa in *A. platanoides*, −1.27 ± 0.05 MPa in *F. sylvatica* and − 1.20 ± 0.06 MPa in *S. nigra*). The pressure was then slowly released from the pressure chamber (to avoid leaf damage) before leaf weight was determined gravimetrically (Sartorius BP61S, 0.0001 g precision, Sartorius AG, Germany). Then, leaves were sprayed with rainwater (collected at the Botanical Garden of the University of Innsbruck) and placed between foam pads already wetted with the same rainwater and enclosed in a plastic box (positioned in a darkened room at 21.2 ± 0.01°C). Petioles and/or axes were not in contact with water. This setting (see setup in Figure S1) enabled sufficient wetting of leaves while they were sufficiently supplied with air and thus mimicked the situation of a rainy day. After 19 h, leaves were carefully dried with paper towels and immediately weighed before Ψ was re‐measured. In *S. nigra*, petiole tips occasionally had to be trimmed (<1 mm) to enable accurate visibility of the rising water column. The leaf water uptake (FWU; mol m^−2^ s^−1^) was calculated as:

(1) $$\mathrm{FWU}=\left(\mathrm{\Delta W}\right)/\left(\mathrm{\Delta t}\cdot \mathrm{LA}\cdot 18.015\right)$$

where ΔW (g) is the gain in weight during the measurement interval Δt (s) and LA (m^2^) is the leaf area. The molecular mass of water (18.015 g mol−^1^) is required for the conversion to mol. LA was determined as described below (see Minimum leaf conductance).

The difference in leaf water potential (ΔΨ) before and after leaf water uptake was also calculated.

### 2.3 Minimum leaf conductance

Saturated leaves were cut from the branches, and their weight determined gravimetrically. Then, leaves were placed on a fine net and ventilated with a fan (wind speed ca., 1 m s^−1^) for dehydration. This occurred in a darkened room to ensure closed stomata. Relative air humidity and temperature were registered at 1 min intervals with an EMS 33 sensor connected to an EMS Edgebox V8 datalogger (EMS Brno, Czech Republic). After about 10, 20, and 30 min, each leaf was re‐weighed, and the leaf transpiration (EV_leaf_; mol m^−2^ s^−1^) was calculated as:

(2) $${\mathrm{EV}}_{\text{leaf}}=\left(\mathrm{\Delta W}\right)/\left(\mathrm{\Delta t}\cdot \mathrm{LA}\cdot 18.015\right)$$

where ΔW (g) is the loss in weight during the measurement interval Δt (s) and LA (m^2^) is the leaf area. The molecular mass of water (18.015 g mol^−1^) is required for the conversion to mol. LA was calculated by scanning leaves and determining their projected area with the image analysis software ImageJ 1.51Q (National Institutes of Health, USA). Please note that the projected leaf area was used in calculations to be consistent with standard gas exchange measurements on hypostomatal leaves, though minimum leaf transpiration and FWU occurred over the entire leaf surface. Minimum leaf conductance *g*_min_ (mol m^−2^ s^−1^) was calculated as:

(3) $$g_{\min}={\mathrm{EV}}_{\text{leaf}}\cdot P/\left(\mathrm{SVP}‐\mathrm{VP}\right)$$

where SVP is the saturated vapor pressure, VP the actual vapor pressure, and P the atmospheric pressure (all in Pa). SVP and VP were calculated from relative air humidity and air temperature following Huang (2018).

### 2.4 Leaf wettability

**FIGURE 1:**
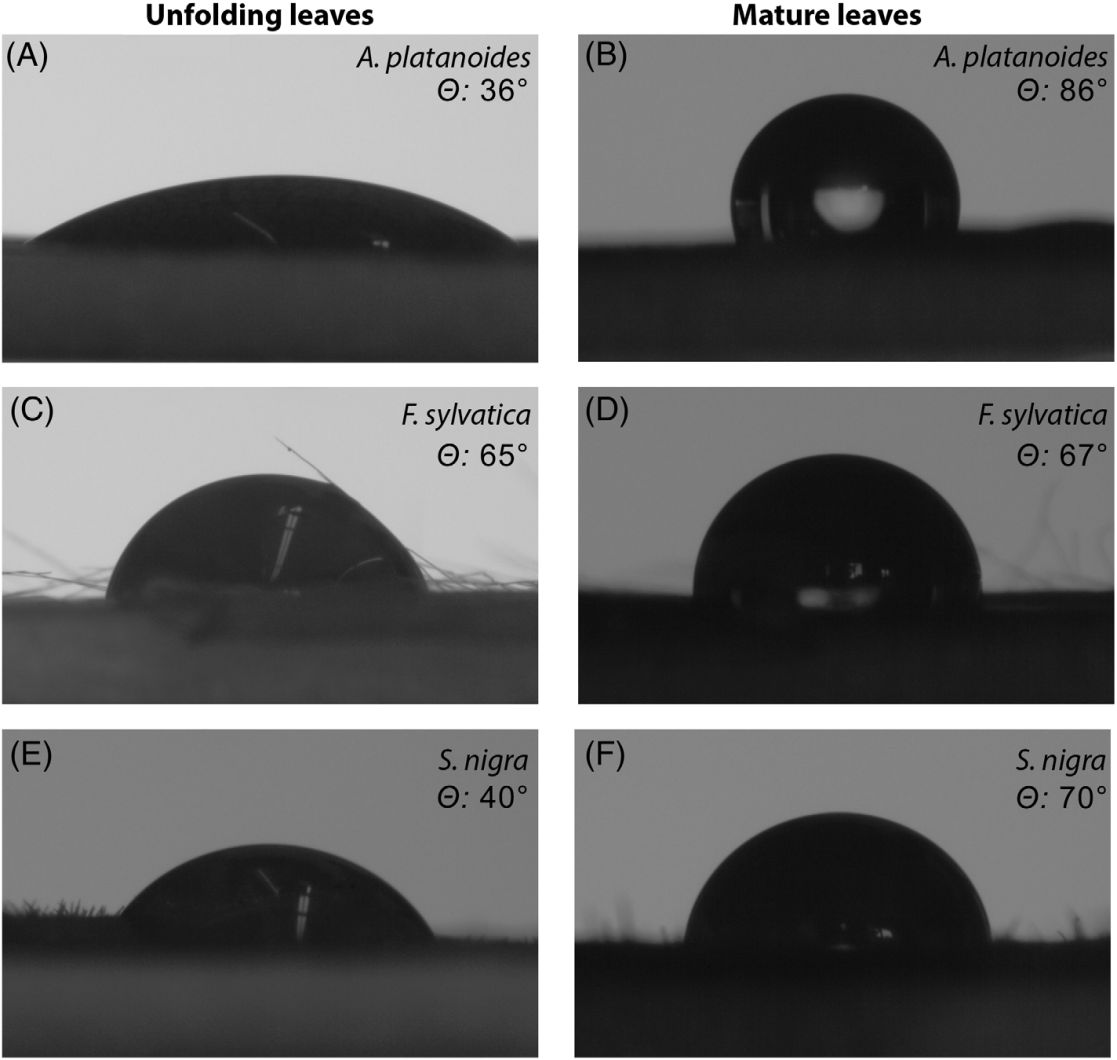
Rainwater droplet (6 μL) deposited on the adaxial surface of unfolding and mature leaves of *A. platanoides* (A–B), *F. sylvatica* (C–D), and *S. nigra* (E–F). Corresponding contact angles θ are also reported.

Leaf wettability was determined by measuring the contact angle (θ) of a 6 μL rainwater droplet deposited on the leaf surface (i.e., sessile drop‐method; see Adam, 1963). Leaves were placed on a microscope slide, and their surface was maintained flat with the help of double‐sided tape. θ measurements were performed with a video‐based optical contact angle measuring system OCA 15EC (DataPhysics Instruments GmbH, Germany). This device enabled us to accurately place a water droplet on top of the leaf surface (see Figure 1), and digital images of the 6 μL rainwater droplets were taken by an integrated digital camera. Images were directly analyzed with the featured SCA software enabling θ calculations.

Contact angle measurements were done on the adaxial (θ_ad_) and abaxial leaf surface (θ_ab_).

θ determined leaf wettability as extreme‐hydrophilic (θ < 40°), highly wettable (40° < θ < 90°), wettable (90° < θ < 110°), nonwettable (110° < θ < 130°), highly nonwettable (130° < θ < 150°) and super‐hydrophobic (150° < θ < 170°; see Aryal & Neuner, 2016).

### 2.5 Statistics

All values are given as mean ± standard error. Differences between leaf stages of the same species were tested using a one‐way ANOVA followed by Tukey's post hoc comparison (FWU, g_min_, ΔΨ, and leaf wettability). For each species, to obtain leaf stages clustering based on traits relationship, we performed a principal component analysis (PCA) from which an evaluation of differentiation between leaf stages was extracted. Correlation analysis between specific parameters was carried out using the Pearson product–moment correlation. All statistical data were analyzed with R 3.6.2 (R Core Team, 2017) at a probability level of 5%.

## 3 RESULTS

### 3.1 Foliar water uptake

**FIGURE 2:**
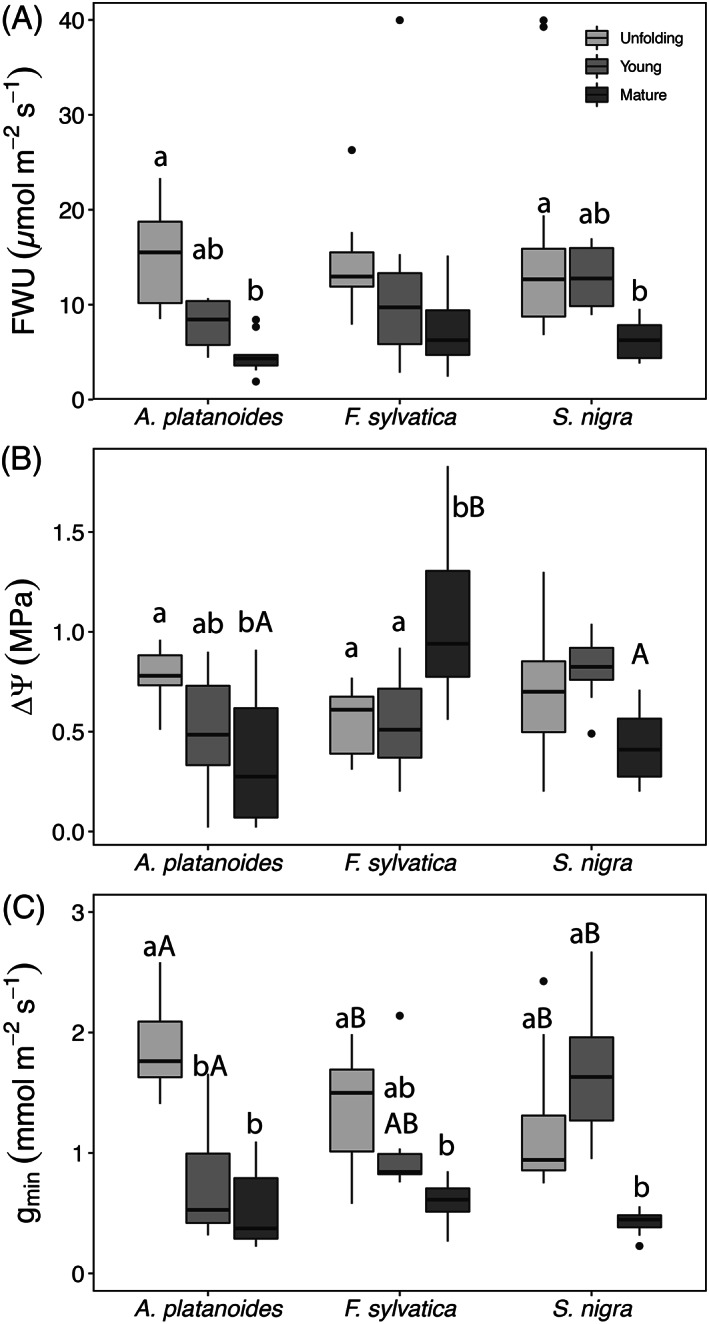
Leaf water uptake FWU (A), change in leaf water potential ΔΨ before and after foliar water uptake (19 h of exposure of leaf blades to wet pads in a closed box; (B), and minimum leaf conductance g_min_ (measured between 10 and 30 min during dehydration; (C) in unfolding, young and mature leaves of *Acer platanoides*, *Fagus sylvatica* and *Sambucus nigra*. Light to dark shades of gray indicate the three leaf stages. Different lower‐case letters indicate significant differences (*p* < 0.05) between different leaf stages within the same species. Different capital letters indicate significant differences between species at the same leaf stage. Mean ± SE. *n* = 6–20.

In all species, FWU progressively decreased from unfolding to mature leaves, with young leaves showing in‐between values (Figure 2a), and no significant differences between species. Significant differences were found in *A. platanoides* (*p* = 0.002) and *S. nigra* (*p* = 0.011) between unfolding (*A. platanoides* 15.37 ± 1.48 μmol m^−2^ s^−1^; *S. nigra* 14.80 ± 2.07 μmol m^−2^ s^−1^) and mature leaves (*A. platanoides* 4.64 ± 0.63 μmol m^−2^ s^−1^; *S. nigra* 6.24 ± 0.64 μmol m^−2^ s^−1^) (Figure 2a).

After 19 h of FWU, younger leaf stages showed significantly higher recovery of leaf Ψ than mature leaves in *A. platanoides* (ΔΨ unfolding 0.78 ± 0.04 MPa; mature 0.34 ± 0.12 MPa; P = 0.009) (Figure 2B). Although nonsignificant, *S. nigra* unfolding leaves (ΔΨ 0.68 ± 0.07 MPa) also recovered more than mature leaves (0.43 ± 0.06 MPa; Figure 2B). In *F. sylvatica*, mature leaves recovery (ΔΨ 1.07 ± 0.10) was significantly higher than of both unfolding (ΔΨ 0.55 ± 0.05 MPa; *p* < 0.01) and young leaves (ΔΨ 0.53 ± 0.06 MPa; *p* < 0.01) (Figure 2b).

### 3.2 Minimum leaf conductance

Minimum leaf conductance (g_min_) was overall similar across species, except for unfolding leaves of *A. platanoides* which exhibited significantly higher values than unfolding leaves of the other two species (*p* < 0.001; Figure 2c). In *A. platanoides*, unfolding leaves had a significantly higher g_min_ (2.5 ± 0.3 mmol m^−2^ s^−1^) than young (0.7 ± 1.2 mmol m^−2^ s^−1^; *p* < 0.001) and mature leaves (0.5 ± 0.1 mmol m^−2^ s^−1^; *p* < 0.01). *F. sylvatica* showed a similar pattern with g_min_ of unfolding leaves (1.4 ± 0.1 mmol m^−2^ s^−1^) being significantly higher than mature leaves only (0.6 ± 0.0 mmol m^−2^ s^−1^; *p* = 0.04), and in *S. nigra*, g_min_ of mature leaves (0.4 ± 0.0 mmol m^−2^ s^−1^) was significantly lower than both unfolding (1.2 ± 0.2 mmol m^−2^ s^−1^; *p* = 0.019) and young leaves (1.7 ± 0.2 mmol m^−2^ s^−1^; *p* < 0.001) (Figure 2c).

### 3.3 Leaf wettability

**FIGURE 3:**
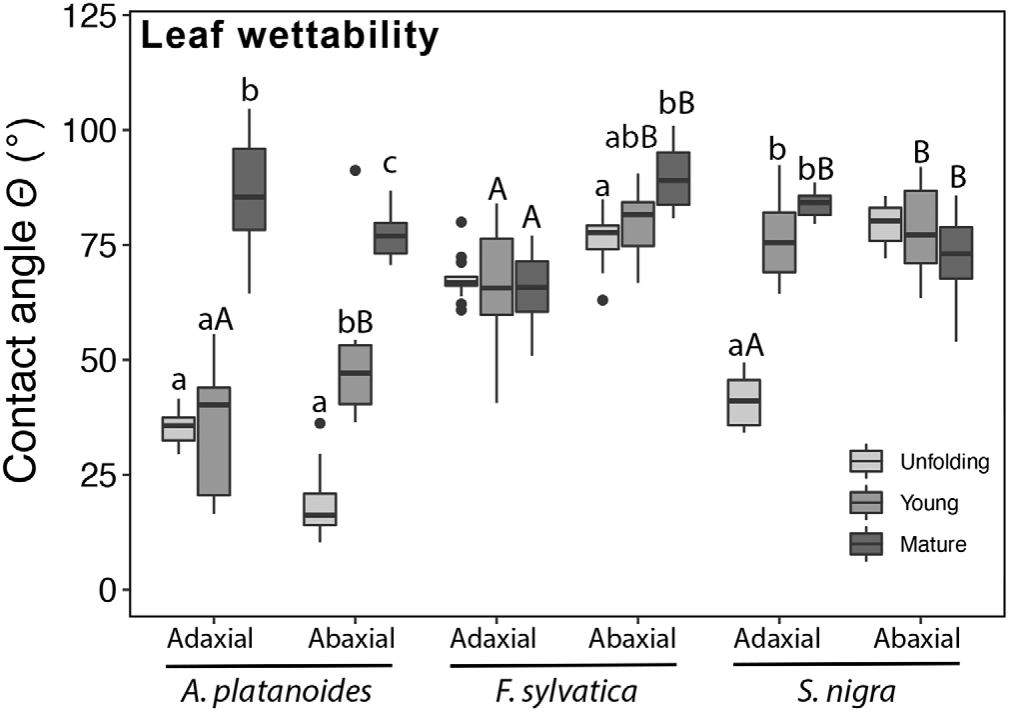
Contact angle θ of rainwater droplets on the adaxial and abaxial surface of unfolding, young and mature leaves of *Acer platanoides*, *Fagus sylvatica*, and *Sambucus nigra*. Different lower‐case letters indicate significant differences (*p* < 0.05) between different leaf stages within the same species. Different capital letters indicate significant differences between the adaxial and abaxial leaf surface at the same leaf stage. Mean ± SE. *n* = 8–16.

In all species under study, a broad range of leaf wettability was observed, and in most cases, wettability decreased during leaf development (Figure 3). Differences in leaf wettability were also found between the adaxial and abaxial surfaces within the same leaf stage (Figures 1 and 3). Contact angles (θ) ranged from a minimum of 18.8 ± 2.6° on the abaxial surface of *A. platanoides* unfolding leaves to a maximum of 89.8 ± 2.0° on the abaxial surface of *F. sylvatica* mature leaves (Figure 3), thus covering a wide range of wettability from extreme‐hydrophilic (θ < 40°) to highly wettable (40° < θ < 90°; see methods). In *A. platanoides*, younger leaf stages were more wettable (θ ca. 35°) than mature leaves (Figure 3), with the adaxial leaf surface of unfolding (θ_ad_ 35.2 ± 1.1°; *p* < 0.001) and young leaves (θ_ad_ 35.3 ± 3.8°; *p* < 0.001) being significantly more wettable than mature leaves (θ_ad_ 86.2 ± 3.8°). Similarly, the adaxial surface of *S. nigra* unfolding leaves (θ_ad_ 40.8 ± 1.2°) was significantly more wettable than that of young (θ_ad_ 76.2 ± 2.4; *p* < 00.1) and mature leaves (θ_ad_ 83.9 ± 1.1; *p* < 00.1), and the abaxial surface of *F. sylvatica* unfolding leaves (θ_ab_ 76.6 ± 1.5) was significantly more wettable than mature leaves (θ_ab_ 89.8 ± 2.0; *p* = 0.002). Overall, no differences in wettability were observed across leaf stages of the adaxial leaf surface of *F. sylvatica* and the abaxial leaf surface of *S. nigra* (Figure 3). Differences in wettability between adaxial and abaxial leaf surfaces of the same species were observed in *F. sylvatica* young and mature leaves and in *A. platanoides* and *S. nigra* young leaves, with the adaxial leaf surface being significantly more wettable (*p* < 0.01 in *F. sylvatica* and *S. nigra* and *p* = 0.0035 in *A. platanoides*), and in *S. nigra* mature leaves, with the abaxial leaf surface being more wettable (*p* = 0.02; Figure 3).

### 3.4 Interrelation of measured parameters

**FIGURE 4:**
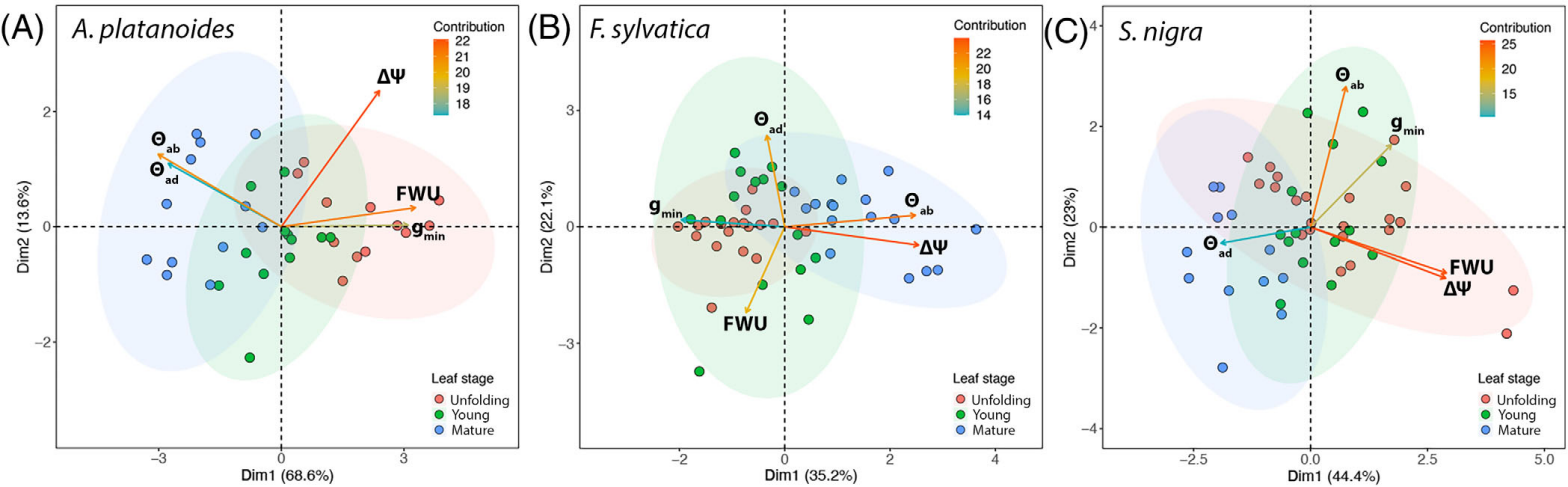
Principal component analysis (PCA) showing the general association among measured leaf traits (minimum leaf conductance g_min_, leaf water uptake FWU, change in leaf water potential ΔΨ, wettability contact angle of the adaxial θ_ad_, and abaxial leaf surface θ_ab_) on the three species under study: *A. platanoides* (A), *F. sylvatica* (B) and *S. nigra* (C). Shaded areas represent cluster grouping of the three different leaf stages (unfolding: red; young: green; mature: blue). The arrow color indicates the contribution in percentage (see color code in legend) of each variable to the principal components.

The relationships among measured parameters between leaf stages within the same species were assessed by performing a PCA (Figure 4). In *A. platanoides*, the first two constructed axes (principal components) explained 82.2% of the variance (Figure 4a). The PCA showed positive relations among FWU and g_min_ (*p* < 0.001), while the opposite vector directions of wettability (both θ_ab_ and θ_ad_) and FWU and g_min_ indicated that an increase in FWU and g_min_ were affected by a decrease in θ (i.e., by higher levels of wettability) (*p* < 0.01). PCA clustering (ellipses) further demonstrated differences between leaf stages, where unfolding leaves (red in Figure 4a) were predominantly associated with higher FWU and g_min_ and higher wettability (i.e., low θ), whereas mature leaves (blue in Figure 4a) were predominantly associated with lower FWU and g_min_ and lower wettability (i.e., high θ). In *F. sylvatica*, the first two constructed axes (principal components) explained 57.3% of the variance (Figure 4b). ΔΨ and θ_ab_ were positively related (*p* < 0.001), while g_min_ was negatively loaded to both, indicating decreasing g_min_ to be related to increasing ΔΨ (*p* = 0.024) and θ_ab_ (Figure 4b). FWU was negatively loaded to θ_ad,_ indicating that an increase in FWU was affected by a decrease in θ_ad_. Clustering indicated differences between mature leaves (blue in Figure 4b) and unfolding and young leaves (red and green in Figure 4b), mature leaves were predominantly associated with increasing ΔΨ and θ_ab_. No difference was observed between *S. nigra* leaf stages (clusters in Figure 4c). The first two constructed axes explained 67.4% of the variance (Figure 4c), and the PCA showed positive relations among FWU and ΔΨ (*p* < 0.001), which were negatively related to θ_ad_, and between g_min_ and θ_ab_.

## 4 DISCUSSION

Studied leaf traits differed between species and leaf stages, with FWU and minimum leaf conductance (g_min_) being higher in the early stages of leaf development. The recovery in leaf water potential (ΔΨ) corresponded to FWU and g_min_ in most cases. A notable exception was *F. sylvatica*, where the recovery in mature leaves was significantly more pronounced, probably supported by water uptake over trichomes. Most leaves were highly wettable (40° < θ < 90°) and no consistent differences were found between adaxial and abaxial leaf surfaces. However, in all studied species, at least one leaf surface showed a decrease in wettability from unfolding to mature leaves, thus indicating higher leaf wettability resulting in higher water absorption in early leaf stages.

### 4.1 FWU, minimum leaf conductance, and water potential

In all species, FWU and g_min_ were highest during the early leaf stages. FWU of unfolding leaves was about two‐fold higher than in mature leaves (Figure 2a), and the highest g_min_ was observed in *A. platanoides* unfolding leaves (Figure 2c). Both aspects are probably determined by the properties of the leaf cuticle, which functions as a barrier to prevent leaf dehydration (Fernández et al., 2017; Schreiber & Riederer, 1996), but may enable water absorption (e.g., Eller et al., 2013). In early leaf stages, the cuticle is thinner and not fully developed (England & Attiwill, 2011), as cuticular wax formation continues throughout leaf expansion (England & Attiwill, 2011; Gratani & Bonito, 2009; Gülz et al., 1991; Varone & Gratani, 2009), and the frequency, size, shape and aperture width of stomata change during leaf ontogeny (England & Attiwill, 2011; Solárová & Pospisilová, 1983). Hence, the combination of a thin, undeveloped cuticle with limited stomatal control might be the reason for both high FWU and g_min_ in younger leaves (Figure 2a,c). In *A. platanoides*, the larger area of leaves than the other species results in a larger transpiring area and might require a more flexible epidermis and cuticle during unfolding, thus resulting in the observed extraordinary high g_min_ (Figure 2c). These results are in agreement with the so‐called “leaky cuticle hypothesis” (Matos et al., 2022), where cuticles are considered to be equally permeable to water in both directions, and that leaves will absorb or lose water depending on driving gradients (i.e., vapor pressure deficit for g_min_, and ΔΨ for K_FWU_). Accordingly, the calculation of the leaf conductivity to the uptake of surface water (k_FWU_; as for Binks et al., 2020) revealed a positive correlation with g_min_ (*p* < 0.001; Figure S2).

Water absorption via the leaves led to a recovery in leaf water potential (ΔΨ) in all studied species. In *A. platanoides* and *S. nigra*, ΔΨ was more pronounced in younger leaves and corresponded to higher FWU and g_min_ (Figure 2). These results are also supported by the PCA, where FWU, g_min_, and ΔΨ were all positively loaded (Figure 4A,C).

Interesting ΔΨ results were found in mature leaves of *F. sylvatica* as they showed remarkably high values (compared to only moderate FWU) (ΔΨ 1.07 ± 0.10 MPa; Figure 2B). As shown by Schreel et al. (2020), the hollow and air‐filled trichomes of *F. sylvatica* leaves absorb water, indicating trichomes to be a major pathway for FWU and rehydration of leaf tissues (see also Schreel et al., 2022). Even scar tissues formed after trichome shedding have been reported to favor water absorption (Fernández et al., 2014). Schreel et al. (2022) showed that water absorbed by *F. sylvatica* leaves entered the hydraulic pathway (following a Ψ gradient) via functional vessels and was redistributed towards lower organs. In the long‐term, this may also support embolism repair in the xylem, as previously observed in *Picea glauca* (Laur & Hacke, 2014). Interestingly, trichomes were also present in *S. nigra* leaves (see Figure 1E,F), but they did not contribute to FWU and leaf Ψ recovery to the same extent as in mature *F. sylvatica* leaves (Figure 2A,B). It is likely that the smaller size and lower density of *S. nigra* trichomes did not enable the effective FWU of *F. sylvatica* leaves (see Figure 1). Hence, although all leaves absorbed water via FWU, trichomes of mature *F. sylvatica* leaves probably supported intracellular transport and thus enabled higher ΔΨ than in other species/leaves under study. *A. platanoides* and *S. nigra* might also exhibit higher leaf hydraulic capacitance than *F. sylvatica*, which resulted in a faster increase of Ψ in the latter.

### 4.2 FWU and wettability

Most leaves under study were highly wettable (40° < θ < 90°), and some even showed extreme‐hydrophilic properties (θ < 40°; see methods and Aryal & Neuner, 2016 for a wettability classification). The glabrous leaves of *A. platanoides* (Figure 1A,B) showed extreme‐hydrophilicity (θ < 40°) in the adaxial side of unfolding and young leaves, and in the abaxial side of unfolding leaves (Figure 3). Even though *F. sylvatica* and *S. nigra* leaves were not as wettable as those of *A. platanoides*, their θ was surprisingly low despite the presence of trichomes. Trichomes may cause hydrophobicity when they are dense and of particular structure and chemical composition, as shown on the abaxial side of *Quercus ilex* leaves (θ > 130°; Fernández et al., 2014). However, Schreel et al. (2020) reported *F. sylvatica* trichomes to be hydrophilic due to the presence of a pectin‐coating and the absence of hydrophobic cutin layers. Trichomes might also stabilize water droplets on the leaf surface (as shown in Figure 1) long enough to keep leaves wet and aid water absorption before droplets could potentially run off (see also Pina et al., 2016).

No consistent difference in wettability was found between the adaxial and abaxial sides, but at least one side (either adaxial or abaxial) of leaves under study showed a significant decrease in wettability from unfolding to mature leaves (Figure 3). Higher leaf wettability probably supports FWU in early leaf stages (Figure 3), which is also reflected by the PCA analysis where FWU and θ (either adaxial or abaxial) were loaded in opposite directions indicating higher rates of FWU corresponding to lower θ (i.e., higher wettability) (Figure 4). PCA analysis also showed g_min_ and θ (either adaxial or abaxial) to be negatively loaded in all three species but positively loaded in *S. nigra* (only for θ_ab_) (Figure 4). This means that higher wettabilities (i.e., low θ), which support FWU, corresponded to overall higher g_min_, especially in unfolding leaves. These results again agree with the “leaky cuticle hypothesis” (Matos et al., 2022; see also above).

## 5 CONCLUSION

FWU in young leaves may enable plants to improve their water status. At the same time, FWU may also correspond with greater water loss, depending on the environmental conditions and the existing water potential gradient across the leaf surfaces. This relatively high conductivity of the immature epidermal and cuticular layers may play an important role during the onset of the growing season when plants are most susceptible to drought stress. With respect to climate change, FWU might gain more importance when the intensity and frequency of spring droughts increase, as already observed in European forests (e.g., Brázdil et al., 2015; Spinoni et al., 2017). Observed high wettability in young developmental leaf stages and species‐specific leaf structures, such as trichomes, may be crucial as they likely support FWU. Future investigations on changes in chemical and structural properties of leaf cuticles in glabrous and pubescent leaves across leaf developmental stages would improve our understanding of FWU and the underlying physiological processes as well as ecological consequences.

## AUTHOR CONTRIBUTIONS

Stefan Mayr and Uwe Hacke planned and designed the present study. Birgit Dämon and Adriano Losso performed experiments and measurements. Adriano Losso performed data analyses. The manuscript was prepared by Adriano Losso with contributions from all other authors.

## Supporting information


**Figure S1.** Experiment setup for the foliar water uptake experiment.
**Figure S2.** Minimum leaf conductance versus leaf conductance to the uptake of surface water.

## Data Availability

All data supporting the findings of this study are available within the paper published online.
