# Supplementary material for: High potential for foliar water uptake in early stages of leaf development of three woody angiosperms
Source: Physiol Plant. 2023 Jul 4;175(4):e13961. doi: 10.1111/ppl.13961 (PMC10953411; doi:10.1111/ppl.13961)
Supplement: Supplementary file 1 — Figure S1. Experiment setup for the foliar water uptake experiment. Figure S2. Minimum leaf conductance versus leaf conductance to the uptake of surface water. [file PPL-175-0-s001.pdf]

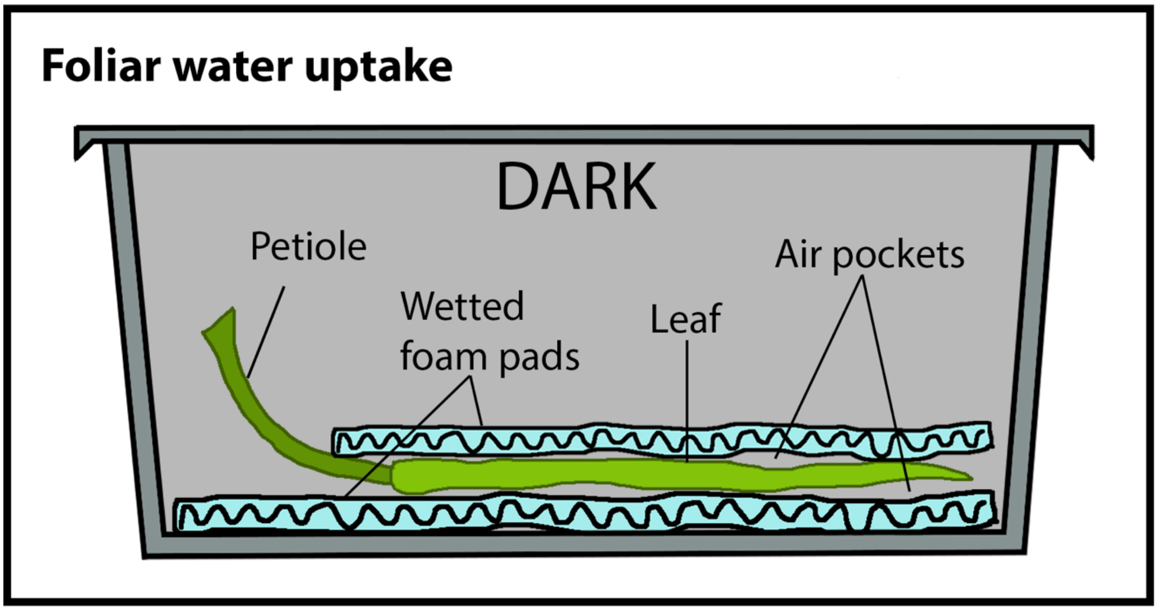

Figure S1. Experiment setup for the foliar water uptake experiment. After being sprayed with rainwater, leaves were placed between two foam pads already wetted with the same rainwater and enclosed in a plastic box (positioned in a darkened room at 21.2 ± 0.01 °C). Petioles and/or axes were not in contact with water. Leaves and foam pads were not in close contact and thus allowed sufficient wetting of leaves while they were sufficiently supplied with air (presence of air pockets), and thus mimicked the situation of a rainy day.

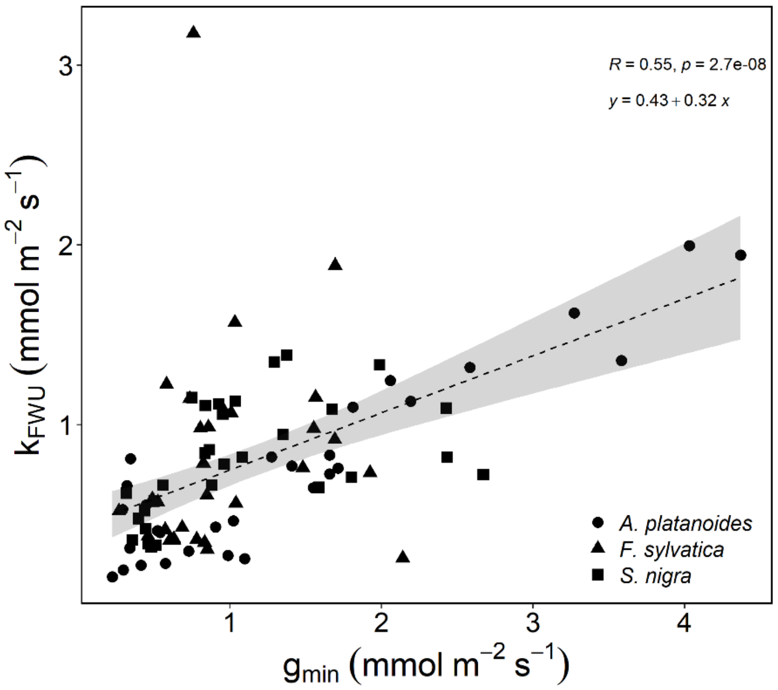

Figure S2. Minimum leaf conductance (g_{min}) versus leaf conductivity to the uptake of surface water (k_{FWU}) of leaves of *A. platanoides* (circles), *F. sylvatica* (tringles) and *S. nigra* (squares). k_{FWU} was calculated based on Binks et al. (2020) as FWU · (P_a / ΔΨ), where P_a is atmospheric pressure. Each point represents a single measurement. Dashed line shows linear regression, and the grey shaded area is its confidence interval.
